# Diagnostic accuracy of the Patient Health Questionnaire 2 (PHQ-2) in Qatar’s primary care settings

**DOI:** 10.1017/S146342362100089X

**Published:** 2022-01-31

**Authors:** Saad Thamer Sedeeq, Mohammad Jamil Mohammad AlTamimi, Ehab Hamed, Mohamed Ahmed Syed

**Affiliations:** 1 Department of Clinical Research, Directorate of Clinical Operations, Qatar University Health Centre, Primary Health Care, Doha, Qatar; 2 Department of Clinical Research, Directorate of Clinical Affairs, Primary Health Care, Doha, Qatar

**Keywords:** depression, Patient Health Questionnaire 2, primary care, Qatar

## Abstract

This cross-sectional study was designed to establish diagnostic accuracy of the Patient Health Questionnaire 2 in Qatar’s primary care population. The data required for the study were anonymously extracted from Qatar’s primary care electronic medical record system. The sensitivity, specificity, predictive values, negative values and optimal cut-off points were calculated for the tool. A total of 6921 individuals met the study’s inclusion criteria. The diagnostic accuracy of cut-off values was calculated for scores 1–6. Based on the Youden’s index (0.58), a score of 2 was identified as the most optimal cut-off. It offers a sensitivity of 88.73% and specificity of 69.31%. Further studies should aim to confirm the results using alternative study designs and to report them in accordance to population characteristics both in Qatar and internationally.

## Background

Globally, depression is the most common psychiatric disorder in the general population (Liu *et al*., 2020). It is a major contributor to the disease burden and a leading cause of disability globally (Wang *et al*., 2017). In primary care settings, depression is the most prevalent mental health condition. Prevalence rates for major depressive disorder range from 3.2% to 27.2% in primary care settings (Craven & Bland, 2013).

Studies have reported under-recognition of depression in primary care (Hirschfeld *et al*., 1997; Fekadu *et al*., 2020). While symptoms are prevalent, primary care patients do not discuss them with their doctors. Barriers to diagnosis include patients’ lack of awareness and understanding of the nature of the disease and its symptoms as well as the variability in clinical presentation. It is estimated that 50% of patients with major depressive disorder are not identified (Mitchell *et al*., 2009). Screening for depression in primary care to provide early identification and intervention is supported by a strong body of evidence (Siniscalchi *et al*., 2020). Given large estimates of underdiagnosed and undertreated depression, routine screening in primary care can improve the detection rate and reduce the disease burden.

Increased use of screening tools can help improve identification and treatment of depression in primary care settings. The most commonly used tool to screen for depression in primary care is the Patient Health Questionnaire (PHQ) (Mitchell *et al*., 2016). There are three main formats of the PHQ: PHQ-9 (linear), PHQ-9 (algorithm) and PHQ-2. PHQ-9 includes nine questions, whereas PHQ-2 includes the first two questions of PHQ-9. It is designed as an initial screening tool to be followed by the more comprehensive PHQ-9 and diagnostic interviews.

In Qatar, the current clinical guidelines recommend the use of PHQ-2 as a screening tool for all patients visiting a primary healthcare setting. If the overall PHQ-2 score is ≥ 3, the comprehensive PHQ-9 and diagnostic interviews are undertaken. This study was designed to establish diagnostic accuracy of the PHQ-2 in Qatar’s primary care population. Its findings will inform local and international guideline development for depression screening in primary healthcare settings.

## Methods

### Study setting

The study was conducted in Primary Health Care Corporation (PHCC) in Qatar. PHCC is a public sector organisation that delivers primary care to approximately 70% of the country’s population through 28 health centres.

### Study population and data collection

PHCC operates a single electronic medical record (EMR) system across all PHCC health centres. The data required for the study were anonymously extracted from PHCC’s EMR system. The eligibility criteria for inclusion were (1) individuals aged > 18 years to <65 years and (2) completed PHQ-2 score in the electronic records between January 2017 and December 2019. Individuals with other mental health conditions (personality disorder, schizophrenia, mental disability and dementia) were excluded.

### Data analysis

Descriptive analysis of age, gender and diagnosis of depression was undertaken. Individuals with no diagnostic codes for depression on the EMR were considered not depressed. A mean PHQ-2 score was calculated. The sensitivity, specificity, predictive values, negative values and optimal cut-off points were calculated for the tool. Youden’s index, area under the curve of a receiver operated curve and gain in certainty metric were calculated to estimate performance.

### Ethical considerations

The study presented a minimal risk of harm to its subjects, and the data collected for it were anonymised. None of the subjects’ personal information was available to the research team. Overall, the study was conducted with integrity according to generally accepted ethical principles and was reviewed and approved under the exempt review category by the PHCC’s research subcommittee (PHCC/DCR/2020/03/017).

## Results

A total of 6921 individuals met the study’s inclusion criteria. The mean age of those included was 40.4 years and 63% were women. Depression was diagnosed in 17.9% of the study population. The mean PHQ-2 score was 1.6.

The diagnostic accuracy of cut-off values was calculated for scores 1–6 (see Table 1). Based on the Youden’s index (0.58), a score of 2 was identified as the most optimal cut-off. It offers a sensitivity of 88.73% and specificity of 69.31%.

**Table 1.** Diagnostic accuracy of PHQ-2 scores by cut-off values

Cut-off point	Sensitivity (%)	Specificity (%)	PPV (%)	NPV (%)	Youden’s index	AUC	Metric score
1	94.44	57.26	32.58	97.92	0.517	0.839	1.52
2	88.73	69.31	38.73	96.57	0.580	0.839	1.58
3	66.51	82.2	44.96	91.82	0.487	0.839	1.49
4	53.95	88.08	49.74	89.74	0.420	0.839	1.42
5	32.69	93.85	53.77	86.44	0.265	0.839	1.27
6	21.18	96.71	58.44	84.87	0.179	0.839	1.18

## Discussion

This study is the first to report diagnostic accuracy of the PHQ-2 in Qatar’s primary care population and potentially in the Gulf Cooperation Countries (which have similar population characteristics as Qatar). Its findings demonstrate the PHQ-2 tool has a high diagnostic accuracy in Qatar’s primary care settings.

The tool was found to be very sensitive for a diagnosis of depression with sensitivities of 95% and 88% for thresholds of ≥ 1 and ≥ 2, respectively. However, it had a modest specificity of 57% and 69%, respectively, at these cut-off values. The finding that a score of ≥ 2 was more successful in detecting cases of depression than the current score ≥ 3 suggests that it may be too high for clinical practice. A systematic review and meta-analysis also concluded that ≥2 may be preferable if clinicians want to ensure that few cases of depression are missed. Another systematic review and meta-analysis reported that the combination of PHQ-2 (with cut-off ≥2) followed by PHQ-9 (with cut-off ≥10) had similar sensitivity but higher specificity compared with PHQ-9 cut-off scores of 10 or greater alone (Levis *et al.*, 2020). Cut-off scores of ≥ 2 are supported by other studies and healthcare settings (Yu *et al*., 2011; Thombs *et al*., 2014; Carey *et al*., 2016; Gelaye *et al*., 2016; Scoppetta *et al*., 2021). Therefore, it is recommended that clinical guidelines are reviewed and revised taking these findings into consideration. Using a higher cut-off value may be a reason for underdiagnosed in primary healthcare settings as reported by previous studies (Mitchell *et al*., 2009).

The study’s strength includes a large sample and reliable data recorded by qualified healthcare professionals and extracted from an EMR system. The study reports an overview of PHQ-2 diagnostic accuracy in Qatar’s primary care settings. This facilitates development of clinical guidelines that can enhance diagnosis of depression. The limitations of the study include the following: a cross-sectional study design which provides a snapshot in time. Furthermore, the study included only patients who were 18 years and above and those who completed a PHQ-2 questionnaire. Also, the clinical diagnosis of depression is subject to diagnostic variability among clinicians.

The study demonstrates the PHQ-2 is most effective with a cut-off score of ≥ 2 in Qatar’s primary care settings. Clinical guidelines in the country should be aligned with the findings. Further studies should aim to confirm the results using alternative study designs and to report them in accordance to population characteristics both in Qatar and internationally.
