# Diagnostic Accuracy of the Patient Health Questionnaire 2 (PHQ-2) in Qatar’s Primary Care Settings – ERRATUM

**DOI:** 10.1017/S1463423622000093

**Published:** 2022-06-08

**Authors:** Saad Thamer Sedeeq, Mohammad Jamil Mohammad AlTamimi, Ehab Hamed, Mohamed Ahmed Syed

The originally published version of this paper was published with incorrect author affiliations listed. This has been corrected and updated in the online article.

The Publishers would like to apologise for this mistake.
